# Modulation of Spontaneous Action Potential Rate by Inositol Trisphosphate in Myocytes from the Rabbit Atrioventricular Node

**DOI:** 10.3390/cells13171455

**Published:** 2024-08-30

**Authors:** Hongwei Cheng, Cherrie H. T. Kong, Andrew F. James, Mark B. Cannell, Jules C. Hancox

**Affiliations:** School of Physiology, Pharmacology and Neuroscience, University of Bristol, Biomedical Sciences Building, University Walk, Bristol BS8 1TD, UK; hongwei.cheng@bristol.ac.uk (H.C.); cherrie.kong@bristol.ac.uk (C.H.T.K.); a.james@bristol.ac.uk (A.F.J.); mark.cannell@bristol.ac.uk (M.B.C.)

**Keywords:** action potential, atrioventricular node, AVN, 2-APB, IP_3_, IP_3_-R, IP_3_-R2, pacemaking, xestospongin C

## Abstract

The atrioventricular node (AVN) is a key component of the cardiac conduction system and takes over pacemaking of the ventricles if the sinoatrial node fails. IP_3_ (inositol 1,4,5 *tris*phosphate) can modulate excitability of myocytes from other regions of the heart, but it is not known whether IP_3_ receptor (IP_3_-R) activation modulates AVN cell pacemaking. Consequently, this study investigated effects of IP_3_ on spontaneous action potentials (APs) from AVN cells isolated from rabbit hearts. Immunohistochemistry and confocal imaging demonstrated the presence of IP_3_-R2 in isolated AVN cells, with partial overlap with RyR2 ryanodine receptors seen in co-labelling experiments. In whole-cell recordings at physiological temperature, application of 10 µM membrane-permeant Bt_3_-(1,4,5)IP_3_-AM accelerated spontaneous AP rate and increased diastolic depolarization rate, without direct effects on I_Ca,L_, I_Kr_, I_f_ or I_NCX_. By contrast, application via the patch pipette of 5 µM of the IP_3_-R inhibitor xestospongin C led to a slowing in spontaneous AP rate and prevented 10 µM Bt_3_-(1,4,5)IP_3_-AM application from increasing the AP rate. UV excitation of AVN cells loaded with caged-IP_3_ led to an acceleration in AP rate, the magnitude of which increased with the extent of UV excitation. 2-APB slowed spontaneous AP rate, consistent with a role for constitutive IP_3_-R activity; however, it was also found to inhibit I_Ca,L_ and I_Kr_, confounding its use for studying IP_3_-R. Under AP voltage clamp, UV excitation of AVN cells loaded with caged IP_3_ activated an inward current during diastolic depolarization. Collectively, these results demonstrate that IP_3_ can modulate AVN cell pacemaking rate.

## 1. Introduction

The atrioventricular node (AVN) is the only pathway in structurally normal hearts for electrical activity to pass from the atria to ventricles [1,2]. Relatively slow conduction through the AVN ensures that atrial contraction is complete before ventricular contraction occurs [3]. During some abnormal cardiac rhythms such as atrial fibrillation (AF), slow AVN conduction limits the number of impulses transmitted to the ventricles [2,4,5]. The AVN also acts as a secondary pacemaker that can take over pacemaking if the primary pacemaker (the sino-atrial node—SAN) fails [2,3,6].

From experiments on myocytes isolated from the AVN of several model species, a number of different ionic conductances have been implicated in the genesis of AVN cell pacemaker activity [6,7,8,9,10]. Among these are the funny current, I_f_ [8,11,12], rapid delayed rectifier, I_Kr_ [6,13,14], L-type calcium current, I_Ca,L_ [7,8,15,16], T-type calcium current, I_Ca,T_, [7,8], background sodium current, I_B,Na_, [17], and in mice the tetrodotoxin-sensitive sodium current, I_Na_ [18,19]. In the SAN, it has been established that a Ca^2+^ ‘clock’ also contributes to generation of spontaneous activity (for reviews, see [20,21]). In respect of the AVN, inhibition of sarcoplasmic reticulum (SR) Ca^2+^ release by ryanodine/thapsigargin has been shown to prolong the cycle length of isolated AVN preparations and AVN-paced hearts [18,22,23]. Further, a functional role for sodium–calcium exchange (NCX) in AVN electrogenicity is supported by experiments wherein NCX activity was reduced/inhibited [24,25,26,27]. Thus, it is likely that spontaneous activity in the AVN is influenced by intracellular Ca^2+^ release from the SR coupled to Ca^2+^ modulation of electrogenesis at the cell surface membrane.

Inositol 1,4,5 *tris*phosphate (IP_3_) is a ubiquitous signalling molecule produced from the hydrolysis of phosphatidylinositol 4,5-bisphosphate (PIP_2_) by phospholipase C (PLC) that translocates from the membrane to the cytoplasm. It is well established that IP_3_ releases Ca^2+^ from intracellular stores via IP_3_ receptors (IP_3_-Rs) [28,29]. Although cardiac myocytes generally express a much higher proportion of RyRs than IP_3_-Rs (~100:1), there is evidence that IP_3_-Rs mediate subsarcolemmal, cytoplasmic, and nuclear Ca^2+^ signalling in the heart (for reviews, see [28,29]). The lower cardiac expression of IP_3_-Rs compared to RyRs does not preclude IP_3_-Rs from playing a role in setting SR Ca^2+^ levels, since RyRs are open for only ~20 ms during the cardiac cycle, while an SR leak via IP_3_-R could be continuous [28]. Moreover, the leak via IP_3_-Rs may be amplified by adjacent RyRs to produce Ca^2+^ sparks [28]. In ventricular myocytes, IP_3_-Rs are both expressed in the perinuclear region, where they are suggested to modulate gene transcription [30,31,32,33], and co-localised with RyRs in the sarcoplasmic reticulum [34,35]. The close apposition of L-type Ca^2+^ channels (LTCCs) and RyRs in ventricular myocyte dyads results in Ca^2+^ release from the SR when LTCCs open: IP_3_-R activation increases dyadic Ca^2+^ fluxes during Ca^2+^ transients and increases the Ca^2+^ spark rate [35]. Further, cross-talk between RyRs and IP_3_-Rs increases in heart failure, which may facilitate arrhythmogenesis [34]. In atrial myocytes, IP_3_-Rs are largely localised with peripheral, junctional SR [30,36], where their activity can impinge on electrical and RyR Ca^2+^ signalling. Furthermore, IP_3_-Rs are central to rhythm control in a variety of non-cardiac tissues and in the spontaneous activity of embryonic cardiomyocytes (for review, see [28]). IP_3_-R stimulation has been associated with abnormal automaticity and spontaneous activity in atrial and pulmonary vein cardiomyocytes [36,37,38,39] and with Ca^2+^ waves in subendocardial Purkinje cells following coronary occlusion [40]. IP_3_-R inhibition has also been reported to inhibit adrenaline-mediated changes in amphibian cardiac pacemaker (sinus venosus) [41]. Notably, a modulatory role for IP_3_-R2 in the SAN has been proposed in mice [28,42] and guinea-pigs [43], where it has been linked to actions of G_q_-coupled receptor agonists (endothelin 1 (ET-1; 43) and phenylephrine [43]).

Quantitative PCR, Western blot, and immunolabelling have shown that mouse SAN and AVN express all three IP_3_-R isoforms [42], with the predominant isoform being IP_3_-R2 [42]—this isoform has the highest IP_3_ affinity [44]. Application of a membrane-permeant form of IP_3_ to mouse SAN increased spontaneous Ca^2+^ transient rate and Ca^2+^ spark frequency, whilst the IP_3_-inhibitor 2-aminoethoxydiphenyl borate (2-APB) decreased Ca^2+^ transient amplitude and rate [42]. Similar effects were not observed in preparations from IP_3_-R2 knock-out mice [42]. Evaluation of tritiated IP_3_ binding to the guinea pig heart found higher binding to the atrioventricular conducting system than to the myocardium [45]. While such findings collectively raise the possibility that IP_3_ may modulate AVN cellular electrophysiology, as yet there are no published data that address the question as to whether IP_3_ influences AVN electrogenesis. Therefore, this study was undertaken to determine whether or not interventions targeting cellular IP_3_ influence AVN spontaneous action potential (AP) rate using a well-established rabbit AVN cell preparation [6,17,46,47].

## 2. Materials and Methods

### 2.1. AVN Cell Isolation

Animal procedures were approved by the Animal Welfare and Ethics Review Board (AWERB) of the University of Bristol (AWERB 21/09/10,5.2.3 and 08/12/15,8.2) and conducted in accordance with UK legislation under consecutive project licences issued by the UK Home Office on 17 May 2011 and 21 April 2016. Adult male New Zealand White rabbits (2–3 kg) were killed humanely and their hearts rapidly excised. The AVN cell isolation method employed here has been described previously [15,46,47]. Briefly, following excision, hearts were cannulated and secured to a Langendorff perfusion apparatus through which a series of isolation solutions were perfused, culminating in a collagenase- and protease-containing perfusate (for details, see [15,46,47]). Following enzyme perfusion, hearts were removed from the cannula and the entire AVN region within the Triangle of Koch was identified using anatomical landmarks and excised for enzymatic and mechanical dispersion of cells [15,46,47]. Isolated cells were stored in Kraftbrühe (KB) solution until experimental use [15,46,48].

### 2.2. Electrophysiological Recording

For electrophysiological experiments, cells suspended in KB solution were placed in a recording chamber mounted on an inverted microscope (Nikon Eclipse TE2000-U, Tokyo, Japan). KB solution was replaced by gradual superfusion of Tyrode’s solution containing (in mM): 140 NaCl, 4 KCl, 2 CaCl_2_, 1 MgCl_2_, 10 glucose, and 5 HEPES (pH 7.4 using NaOH). Patch pipettes were pulled from borosilicate glass (AM Systems Inc., Sequim, WA, USA) and heat-polished to resistances of 2–3 MΩ. Pipettes were filled with a solution containing (in mM): 110 KCl, 10 NaCl, 10 HEPES, 0.4 MgCl_2_, 5 glucose, 5 K_2_ATP, and 0.5 Tris-GTP (pH 7.1 with KOH) [49,50]. This solution was used for all action potential (AP) recordings. Maximum diastolic potential (MDP) was taken to be the most negative membrane potential following AP repolarization, and the slope of the diastolic depolarization was determined to be the average slope between the MDP and inflection point that marked the commencement of the AP upstroke. For recording I_Ca,_ I_Kr_, and I_f_, 5 mM K_4_BAPTA was also included in this pipette solution [49,50]. For selective recording of sodium–calcium exchange (NCX) current (I_NCX_), the pipette solution contained (in mM) 110 CsCl, 10 NaCl, 0.4 MgCl_2_, 1 CaCl_2_, 5 EGTA, 10 HEPES, 5 glucose, and 20 TEACl (pH 7.2 with CsOH) [51]. The external solution for I_NCX_ recording was potassium-free Tyrode’s solution containing 10 μM nitrendipine (to inhibit L-type calcium current) and 10 μM strophanthidin (to inhibit the Na^+^/K^+^ pump) [26,51]. Recordings were initiated with bath superfusion of cells. Once the whole-cell configuration had been obtained, control and test solutions were applied at 35–37 °C to the cell under investigation using a home-built, rapid solution exchange device [52]. All electrophysiology recordings were made using an Axopatch 1D amplifier (Molecular Devices, Sunnyvale, CA, USA). Protocols were devised and applied using pClamp 10.3 software (Molecular Devices, Sunnyvale, CA, USA) via a Digidata 1322 (Molecular Devices, Sunnyvale, CA, USA) analogue-to-digital converter. Membrane currents were digitised at 10 kHz, with an appropriate bandwidth on the recording amplifier, whilst for APs, a digitization frequency of 2 kHz was used (cf. [50]).

### 2.3. Calcium Imaging

Most experiments in this study did not employ a Ca^2+^ fluorophore in order to focus on AP recording in the absence of a potential [Ca^2+^]_i_ buffer. However, in one series of experiments (to investigate effects of Bt_3_-(1,4,5)IP_3_-AM), spontaneous Ca^2+^_i_ transients were measured. For these, AVN myocytes were incubated in 2 µM Fluo-4 AM (Invitrogen, Paisley, UK; Cat No. F23917) at room temperature for 20–25 min, followed by replacement with normal Tyrode’s solution for 30 min for de-esterification. Cells were then placed in the recording chamber on the stage of an LSM Pascal confocal microscope (Carl Zeiss, Jena, Germany). Fluo-4 was excited at 488 nm, with emitted fluorescence >505 nm [27]. Line-scan images across the width of spontaneously beating AVN cells were obtained first in normal Tyrode’s solution and then following application of Bt_3_-(1,4,5)IP_3_-AM. All confocal recordings were made at 37 °C. ImageJ (National Institutes of Health, Bethesda, MD, USA) was used for analysis.

### 2.4. Flash Photolysis

Caged-IP_3_ (Sichem, Bremen, Germany. Cat No. cag-6-145) was included in the pipette solution. IP_3_ was released from caged-IP_3_ by UV flash photolysis using an OptoFlash (LED 365 nm; Cairn, Faversham, Kent, UK), which was fitted onto the microscope of patch-clamping recordings. The UV flash duration was 100 ms, and intensity was 1.1 A. Single or multiple short series of flashes (up to 5) were triggered manually (about 1 s for each flash), whilst continuous repeated stimulation was automatically applied with a frequency of 1.3 Hz.

### 2.5. Immunohistochemistry

AVN cells were fixed with 4% paraformaldehyde in phosphate-buffered saline (PBS) for 10 min, permeabilised with 0.1% Triton X-100 for 10 min, then blocked with 10% goat serum for 1 h at room temperature. The cells were then incubated overnight at 4 °C with 2% goat serum containing primary antibodies of rabbit anti-IP_3_-R2 (1:25; Alomone labs, Jerusalem, Israel, Cat No. ACC-116) and mouse anti-RyR2 (1:100; Invitrogen, Paisley, UK; Cat No. MA3-916) or no primary antibody as negative control. Secondary antibodies were incubated for 2 h at room temperature with goat anti-rabbit Alexa Fluor 488 (1:200; Invitrogen, Cat No. A-11008) and goat anti-mouse Alexa Fluor 594 (1:200; Invitrogen, Cat No. A-11005). The cells were mounted in Vectashield mounting medium (Vector Laboratories, Newark, CA, USA) on slides. The IP_3_-R- and RyR-labelled AVN cells were imaged with an LSM880 confocal microscope (Carl Zeiss, Jena, Germany) using a 63× (NA 1.4, oil immersion) objective sampled at ~0.15 μm/pixel and z-stacks (~0.4 μm step size) obtained. Signals were detected at 600–650 nm under 594 nm laser illumination, followed by detection at 495–550 nm under 488 nm laser illumination. Confocal images were deconvolved using the Richardson–Lucy algorithm, with fast Fourier transform (FFT), Pearson, and Mander analyses performed in MATLAB (R2023a, Mathworks, Natick, MA, USA).

### 2.6. Experimental Compounds

Bt_3_-(1,4,5)IP_3_-AM (Sichem, Bremen, Germany, Cat No. 3-1-145) was dissolved in DMSO to produce a stock solution of 10 mM. In order to avoid potential confounding effects of switching artefacts and to allow some time for intracellular cleavage of the ester bond, effects of Bt_3_-(1,4,5)IP_3_-AM were evaluated after at least 110 s of its application. 2-Aminoethyl diphenylborinate (2-APB, Sigma-Aldrich, Gillingham, UK, Cat No. D9754) was dissolved in DMSO to produce stock solutions of 10 and 1 mM. Xestospongin C (Xe-C, Tocris, Bristol, UK, Cat No. 1280) was dissolved in DMSO to produce a stock solution of 0.5 mM (100× stock).

### 2.7. Data Analysis and Statistics

Action potential and current analysis was performed using Clampfit 10.3 software (Molecular Devices, San Jose, CA, USA). Statistical analysis was performed using Microsoft Office 2016 (Professional edition) and Microsoft 365 (Version 2406) Excel (Microsoft Corporation, Redmond, WA, USA) and GraphPad Prism 7.0 and 10.2.0 (GraphPad Software, Boston, MA, USA). All data are expressed as means ± SEM. Statistical comparisons were made using two-sample paired, unpaired *t*-tests, one-sample *t*-tests, or ANOVA (or non-parametric equivalent) as appropriate. *p* < 0.05 was taken as statistically significant.

## 3. Results

### 3.1. Immunohistochemistry

**Figure 1:**
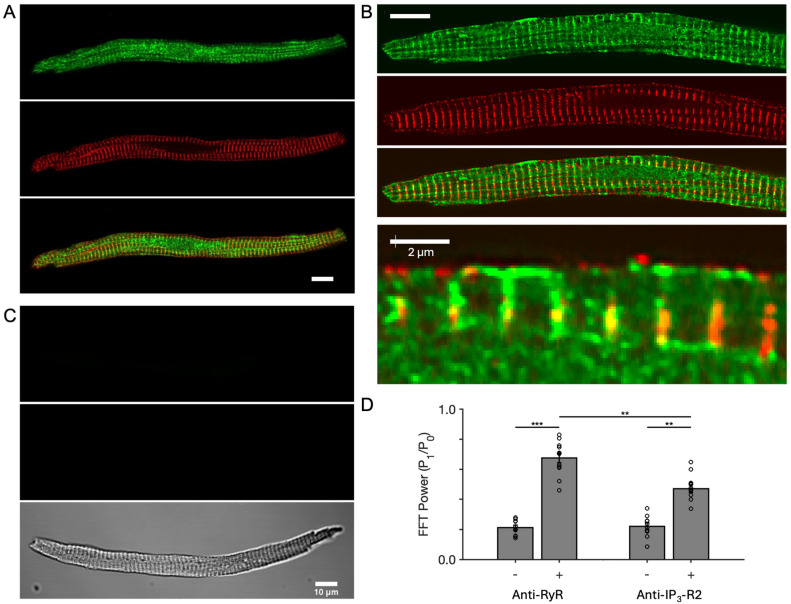
Expression of IP_3_-R2 and co-labelling with RyR2. (**A**) Labelling of a “spindle” shaped isolated AVN cell with (from top to bottom): anti-IP_3_-R2 antibody, anti-RyR2 antibody and a merged image, showing partial overlap of IP_3_-R2 and RyR2 labelling. (**B**) Labelling of a ‘rod’-shaped isolated AVN cell with panels as described for A. The additional bottom panel is a magnified region to show labelling at the perinucleus and sarcolemma. (**C**) Negative controls for IP_3_-R2 (top panel) and RyR2 (middle panel) labelling alongside transmitted light image (bottom panel). Scale bars for A–C 10 µm, unless otherwise stated. (**D**) Regularity of RyR2 and IP_3_-R2 labelling (n = 12) and respective controls (n = 10), as quantified by FFT power (P_1_/P_0_) at ~1.8 µm periodicity. RyR2 = 0.68 ± 0.03 (control = 0.21 ± 0.02), IP_3_-R2 = 0.47 ± 0.03 (control = 0.22 ± 0.02). Comparisons were made using a paired Wilcoxon signed-rank test for RyR2 vs. IP_3_-R2 and Mann–Whitney U test vs. control: ** *p* < 0.01, *** *p* < 0.001.

Immunocytochemistry was carried out using IP_3_-R2 and RyR2 antibodies and cell imaging using confocal microscopy. Figure 1 shows representative co-labelling with anti-IP_3_-R and RyR antibodies and also negative controls. IP_3_-R labelling is evident in transverse bands and at the cell periphery. Consistent with prior data [27], RyR labelling was also observed in transverse bands. The images in Figure 1A,B show partial overlap of IP_3_-R and RyR staining, where a magnified view in Figure 1B shows IP_3_-R labelling near the sarcolemma, RyRs, and nuclear envelope regions. Regularity of RyR and IP_3_-R labelling was quantified using Fourier analysis, for which spectral power at the first harmonic (~1.8 µm) is shown plotted in Figure 1D. For RyR2, this was 0.68 ± 0.03 (control = 0.21 ± 0.02), and for IP_3_-R2, this was 0.47 ± 0.03 (control = 0.22 ± 0.02). Co-localisation analysis yielded a Pearson’s correlation coefficient of 0.31 ± 0.03 (for control images 0.20 ± 0.02; *p* < 0.05, Wilcoxon’s rank sum). The Manders coefficient for RyR labelling overlap with IP_3_-R was 0.31 ± 0.03, and for IP_3_-R overlap with RyRs, it was 0.22 ± 0.03. In summary, rabbit AVN cells contain IP_3_-Rs, exhibiting a partial labelling overlap with RyRs.

### 3.2. Effects of Cell Permeant IP_3_: Bt_3_-(1,4,5)IP_3_-AM

**Figure 2:**
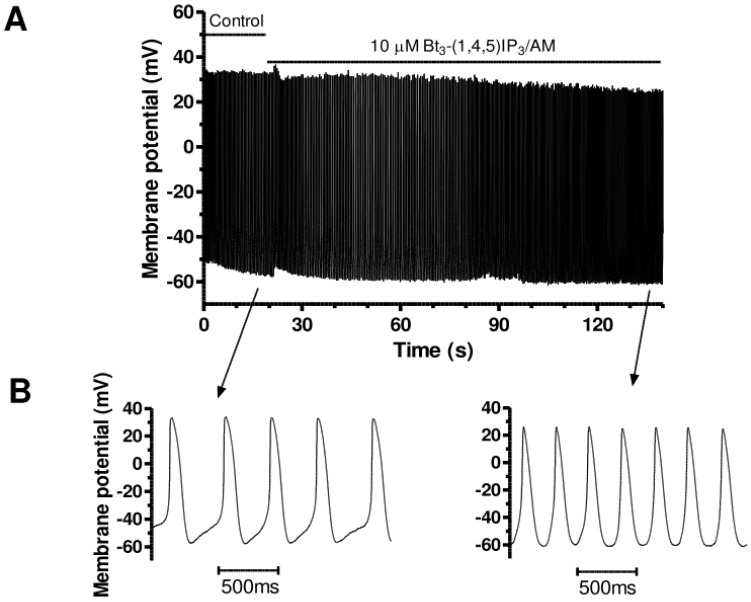
Effects of Bt_3_-(1,4,5)IP_3_-AM on AVN cell spontaneous APs. (**A**) Slow time-base recording (over ~120 s) of spontaneous APs before and during exposure to 10 µM Bt_3_-(1,4,5)IP_3_-AM. (**B**) Faster time-base extracts from the same experiment, showing APs in control (left) and in the presence (right) of 10 μM Bt_3_-(1,4,5)IP_3_-AM (after ~115 s of application, indicated by arrow). Mean AP parameters are given in Table 1.

Membrane permeant esters of IP_3_ have been devised [53] and are known to increase ventricular cardiomyocyte [Ca^2+^]_i_ (e.g., [54]) and to accelerate spontaneous AP rate in murine SAN myocytes [42]. We therefore utilised this approach to determine the response of AVN cells to exogenously applied IP_3_. Figure 2 shows spontaneous APs from an AVN cell before and during exposure to 10 µM Bt_3_-(1,4,5)IP_3_-AM. In the example shown, following a brief switching artefact, spontaneous AP rate increased progressively over ~2 min in the presence of Bt_3_-(1,4,5)IP_3_-AM. This is particularly visible in the expanded time-base records shown in the lower panels of Figure 2 and mean data summarised in Table 1. Spontaneous AP rate was increased by 40.5 ± 5.7% (n = 10, *p* < 0.01) and the slope of diastolic depolarization was significantly increased (from 64.6 ± 8.4 mV s^−1^ to 102.3 ± 10.6 mV s^−1^; n = 10, *p* < 0.05). No significant differences were seen in maximal upstroke velocity, maximal repolarization velocity, or APD_50_ (see Table 1). Despite a tendency for AP overshoot to be reduced following Bt_3_-(1,4,5)IP_3_-AM exposure, the overall reduction in AP amplitude did not attain statistical significance (Table 1).

**Figure 3:**
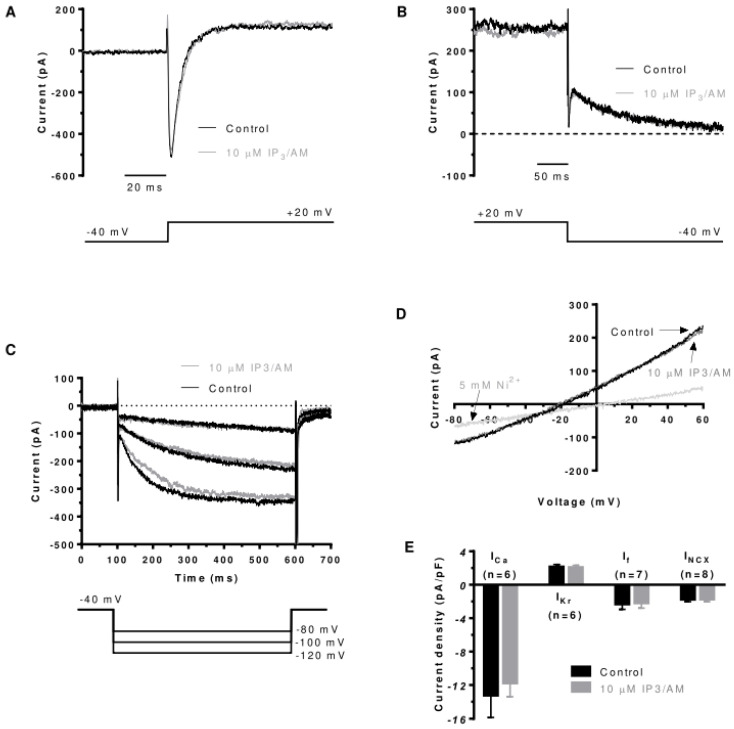
Effects of Bt_3_-(1,4,5)IP_3_-AM on I_Ca,L_, I_Kr_, I_f_ and I_NCX_. (**A**) Representative recordings of I_Ca,L_ (upper) elicited by activating command to +20 mV (lower), in control solution, and 2 min after application of 10 μM Bt_3_-(1,4,5)IP_3_-AM. (**B**) Representative recordings of I_Kr_ tails (upper) following activating command to +20 mV (lower), in control solution, and 2 min after application of 10 µM Bt_3_-(1,4,5)IP_3_-AM. (**C**) I_f_ (time-dependent inward ‘funny’ current; upper traces) elicited by 500 ms hyperpolarizing voltage commands (lower traces) from −40 mV to −120, −100 and −80 mV. (**D**) Current elicited by voltage ramp from +60 to −80 mV under I_NCX_-selective recording conditions in control solution and after 2 min of Bt_3_-(1,4,5)IP_3_-AM application. D also shows inhibition of I_NCX_ by 5 mM Ni^2+^. (**E**) Bar charts illustrating no significant direct effect of Bt_3_-(1,4,5)IP_3_-AM on I_Ca,L_ at +20 mV (n = 6), I_Kr_ tails at −40 mV (n = 6), I_f_ at −120 mV (n = 7) and I_NCX_ at −60 mV (n = 8). Pairwise comparisons for each current were made using *t*-tests.

Bt_3_-(1,4,5)IP_3_-AM was also applied in voltage clamp experiments in which I_Ca,L_, I_Kr_ and the ‘funny’ current I_f_ were measured (see Methods). Figure 3A shows closely superimposed records of I_Ca,L_ in control and Bt_3_-(1,4,5)IP_3_-AM. Figure 3B shows overlaid current records at the end of the +20 mV command and on repolarization to −40 mV. The deactivating tail current at −40 mV shown in Figure 3B has been demonstrated previously to represent AVN I_Kr_, as it is abolished by the selective I_Kr_ inhibitor E-4031, with little evidence for the slow delayed rectifier current, I_Ks_, in rabbit AVN cells [13,46,47]. Bt_3_-(1,4,5)IP_3_-AM did not alter I_Kr_ tail amplitude. Figure 3C shows currents elicited on hyperpolarization to more negative voltages from the holding potential of −40 mV, showing little effect of Bt_3_-(1,4,5)IP_3_-AM on the time-dependent inward current, I_f_. Mean data for I_CaL_, I_Kr_ and I_f_ are shown in Figure 3E, demonstrating no significant effect of Bt_3_-(1,4,5)IP_3_-AM on the three currents. Figure 3D shows currents elicited by a descending voltage ramp protocol that was applied under selective recording conditions for I_NCX_. Again, currents in control solution and in Bt_3_-(1,4,5)IP_3_-AM were found to be closely superimposed. 5 mM Ni^2+^ was applied at the end of the recording period to confirm the identity of the measured current as I_NCX_, with only a small residual Ni^2+^-insensitive current visible. No change in this I_NCX_ was observed following Bt_3_-(1,4,5)IP_3_-AM (Figure 3D,E). Consequently, the dominant effect of Bt_3_-(1,4,5)IP_3_-AM exposure was an acceleration in spontaneous AP rate, without confounding *direct* effects on I_Kr_, I_Ca,L_, I_f_ or I_NCX_.

**Figure 4:**
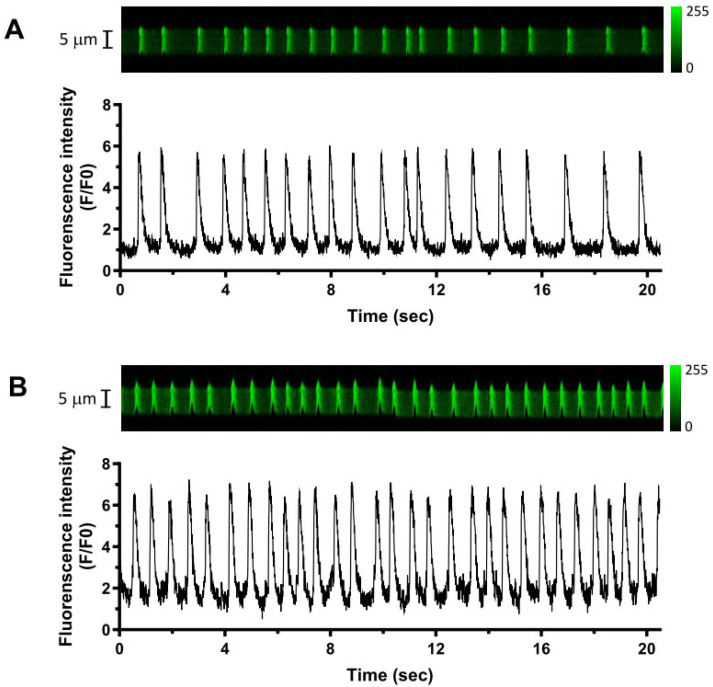
Effects of Bt_3_-(1,4,5)IP_3_-AM on spontaneous Ca^2+^_i_ transients. (**A**) Upper panel shows representative confocal line-scan image of spontaneous [Ca^2+^]_i_ transients in control. Lower panel shows spatially averaged [Ca^2+^]_i_ transient fluorescence. (**B**) Upper panel shows representative confocal line-scan image of spontaneous [Ca^2+^]_i_ transients after 2 min of 10 µM Bt_3_-(1,4,5)IP_3_-AM application. Lower panel shows the spatially averaged fluorescence plot.

To complement the AP experiments, measurements of spontaneous Ca^2+^_i_ transients were made from Fluo-4 loaded undialysed AVN cells (Figure 4; panel A shows control records, while panel B shows records following application of Bt_3_-(1,4,5)IP_3_-AM). The control spontaneous Ca^2+^_i_ transient rate (1.34 ± 0.19 Hz; n = 7) was slower than those for spontaneous AP recordings shown in Table 1 and Figure 2, possibly as a result of Ca^2+^_i_ buffering by the Ca^2+^ fluorophore used for these experiments. Nevertheless, spontaneous rate increased (to 1.78 ± 0.2 Hz; n = 7, *p* < 0.05) following Bt_3_-(1,4,5)IP_3_-AM exposure. Over the time of laser scanning in the continuous presence of Bt_3_-(1,4,5)IP_3_-AM, there was a small upward shift in diastolic fluorescence that overall was not statistically significant (by 16.6 ± 10.0%, *p* > 0.05, n = 7). Ca^2+^_i_ transient amplitude did not change significantly (F/F0: 2.25 ± 0.56 in control, and 2.13 ± 0.60 with Bt_3_-(1,4,5)IP_3_-AM; *p* > 0.05, n = 7), nor did the peak of the Ca^2+^_i_ transient (F/F0: 3.25 ± 0.56 in control, and 3.28 ± 0.70 with Bt_3_-(1,4,5)IP_3_-AM; *p* > 0.05, n = 7).

### 3.3. IP_3_-R Inhibition with Xestospongin C

**Figure 5:**
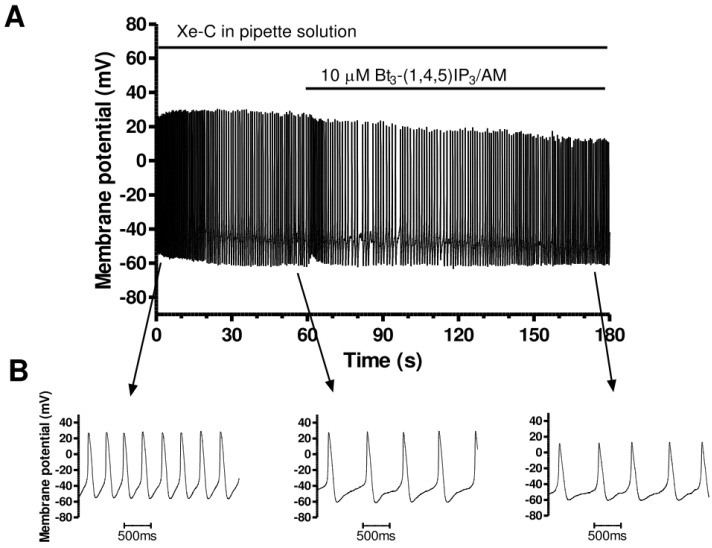
Effect of xestospongin C (Xe-C) on AVN spontaneous AP rate. (**A**) Trace shows a slow time-base recording of spontaneous APs. Horizontal bars indicate periods of 5 μM Xe-C in pipette solution (continuously) and subsequent external application of 10 µM Bt_3_-(1,4,5)IP_3_-AM. (**B**) Faster time-base extracts from the experiment showing 3 time points: at start of recording, after ~1 min recording, and after 2 min of subsequent Bt_3_-(1,4,5)IP_3_-AM application.

We proceeded to determine effects on spontaneous AP generation of the IP_3_-R inhibitor xestospongin C (XeC; [55,56,57]). AVN cells were incubated in 5 µM XeC at room temperature for 1 h and 5 µM XeC was also included in the patch pipette solution. Figure 5 shows exemplar results. AP rate was evaluated immediately on commencing recording (within 30–60 s of attaining the whole-cell patch-clamp configuration) and monitored with time. After 1 min of recording, the spontaneous rate was reduced compared to that at commencement of recording. We then applied 10 µM Bt_3_-(1,4,5)IP_3_-AM, which in the absence of XeC increased spontaneous AP rate (Figure 2, Table 1). As shown in Figure 5, Bt_3_-(1,4,5)IP_3_-AM failed to increase AP rate following prior XeC treatment. Table 2 shows summary data from a total of 11 similar experiments, also including AP parameters for untreated cells (same as control data in Table 1). AP rate and slope of diastolic depolarization after 1 min of recording were significantly less than measured at the start of recording. Comparison with AP parameters from untreated cells (Table 2) suggests that it was inclusion of XeC in the pipette solution rather than pre-incubation in external solution that was important for the inhibitory action of XeC on AP rate to be observed. While there was a trend for diastolic depolarization and AP upstroke velocity to be faster at the start of recording in XeC than in untreated cells, this did not attain statistical significance (*p* > 0.05 for both). In eight experiments in which Bt_3_-(1,4,5)IP_3_-AM was applied in the presence of XeC, there was no significant acceleration in AP rate (2.27 ± 0.25 Hz following Bt_3_-(1,4,5)IP_3_-AM compared to 1.94 ± 0.17 Hz in XeC; *p* = 0.27).

### 3.4. Effect of Photoreleased IP_3_ on AP Rate

**Figure 6:**
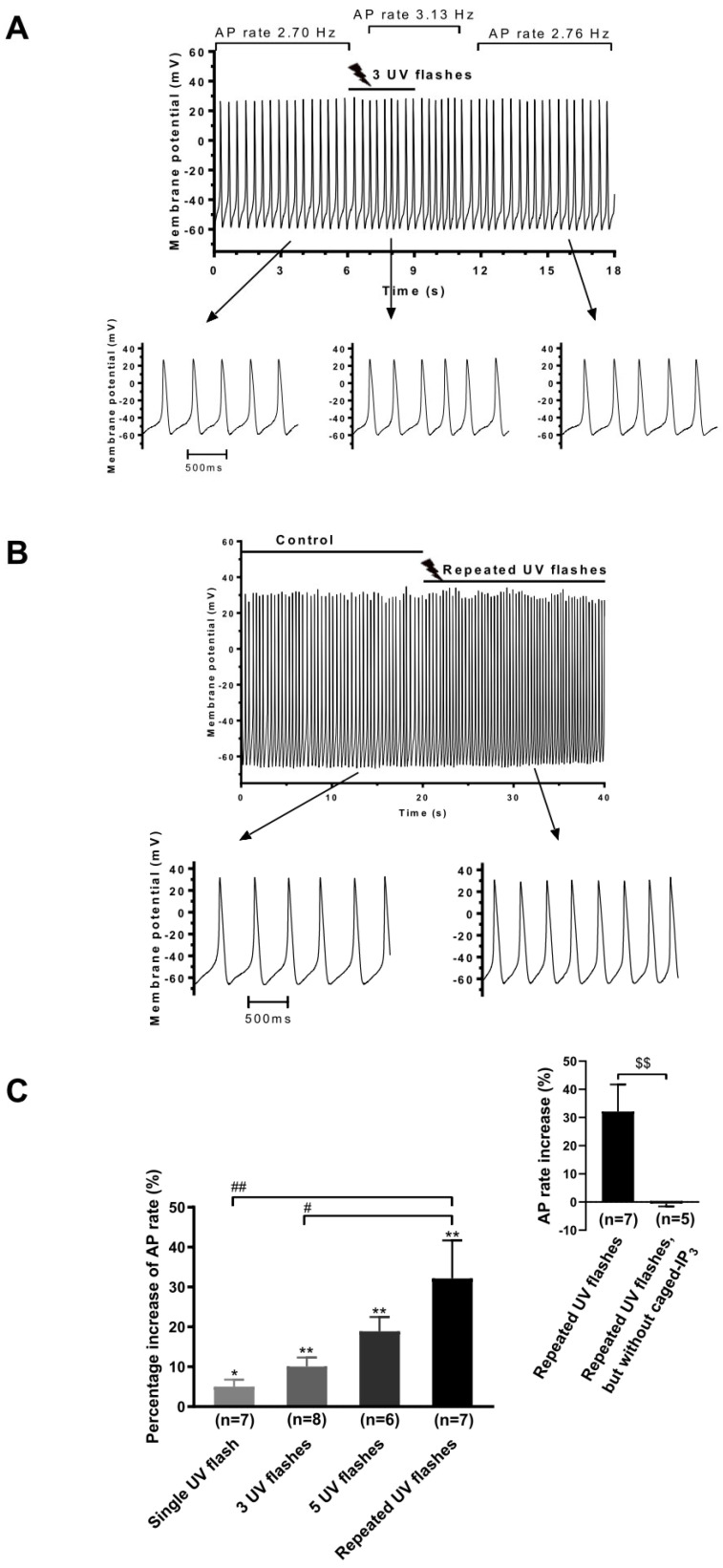
UV flash photolysis of caged IP_3_ increases spontaneous AP rate in rabbit AVN cells. (**A**) Recording showing that UV flash photolysis of caged IP_3_ (three UV flashes over 3 s) transiently increased the spontaneous AP rate. The upper panel displays a slow time-base recording of spontaneous APs. Horizontal bars indicate application of three UV flashes and AP rates before and after the applied UV flash. Lower panels show faster time-base extracts from the experiment, which illustrate clearly the increase in AP rate when IP_3_ was released by UV flash photolysis (middle) compared to control (left). (**B**) Effect of repeated UV flash photolysis of caged-IP_3_ on spontaneous AP rate. Upper panel displays a slow time-base recording of spontaneous APs before and during repeated UV flashes. Lower panels show faster time-base extracts from the experiment, showing increase in AP rate by UV flash (right) compared to control (left). (**C**) Summary of the effect of UV flash photolysis of caged IP_3_ by single, multiple, and repeated UV flashes on spontaneous AP rate in rabbit AVN cells. * *p* < 0.05, ** *p* < 0.01: one sample *t*-test compared with 0. ^#^ *p* < 0.05, ^##^ *p* < 0.01: one-way ANOVA with Bonferroni post hoc comparison. Inset shows comparison of AP rate increase by repeated UV flashes with and without caged IP_3_ (^$$^ *p* < 0.01, unpaired *t*-test). The UV flash itself without caged IP_3_ did not affect AP rate (*p* > 0.05, one sample *t*-test compared with 0). Cell numbers are indicated in parentheses.

In a separate series of experiments, 100 µM caged IP_3_ was introduced into cells via the pipette solution and was uncaged (to release IP_3_) by UV flash photolysis. In the caged state, IP_3_ is biologically inactive until photoreleased by exposure to UV light. We applied single, three, five, and repeated flashes of UV light in these experiments. Figure 6A shows that three successive flashes over ~3 s resulted in a transient, reversible acceleration in spontaneous AP rate. Figure 6B shows the cumulative effect of repeated UV flashes applied over approximately 20 s: with increasing numbers of flashes, the increase in spontaneous AP rate became larger. Figure 6C summarises mean results using differing extents of photostimulation: the extent of acceleration of AVN cell spontaneous AP rate increased as the extent of photostimulation was increased. This clearly demonstrates that exposure to one, three, and five flashes only partially and progressively photolysed caged IP_3_. Importantly, in cells that were not loaded with caged IP_3_, repeated application of UV flashes did not increase spontaneous AP rate (Figure 6C inset), indicating that the presence of caged IP_3_ was required for UV stimulation to result in increased AVN cell AP rate. Table 3 summarises the effects of repeated UV flash stimulation of caged IP_3_. Of the AP parameters shown, only spontaneous rate and slope of diastolic depolarization were significantly increased by photoreleased IP_3_.

### 3.5. Effects of 2-APB

**Figure 7:**
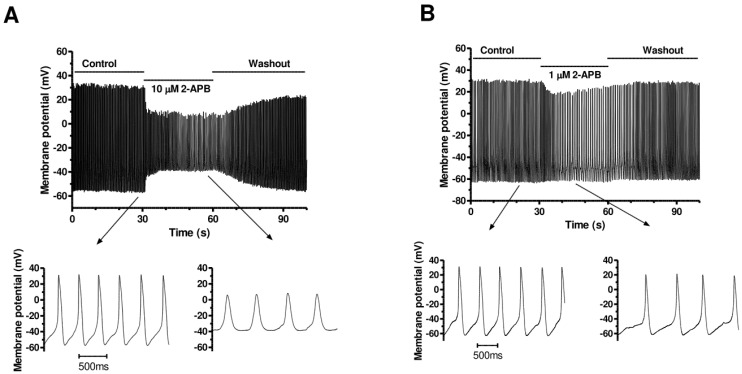
Effects of 2-APB on AVN spontaneous APs. (**A**) Upper panel shows a slow time-base recording (over a period of 100 s) showing the effect of rapid application of 10 µM 2-APB to a spontaneously active AVN cell. Lower panel shows faster time-base extract of APs in control (left) and in the presence (right) of 10 µM 2-APB. (**B**) Upper panel shows a slow time-base recording (over a period of 100 s) showing the effect of rapid application of 1 µM 2-APB to a spontaneously active AVN cell. Lower panel shows faster time-base extract of APs in control (left) and in the presence (right) of 1 µM 2-APB. Mean AP parameters are given in Table 4.

2-Aminoethoxydiphenyl borate (2-APB) exerts an inhibitory effect on constitutively active IP_3_-Rs [58,59,60] and has been utilised in the study of IP_3_-Rs in the SAN [42,43]. In order to investigate whether constitutively active IP_3_-Rs may influence AVN cell rate, we applied 2-APB to spontaneously active AVN cells at two concentrations (10 μM and 1 µM). Representative results are shown in Figure 7A,B. At 10 μM, application of 2-APB rapidly led to a reduction in spontaneous AP rate, accompanied by marked depolarization of the maximal diastolic potential (MDP) and reduction in AP amplitude. Mean AP parameter data from these experiments are shown in Table 4, showing statistically significant decreases in rate (by 28.0 ± 4.3%; n = 7, *p* < 0.01), MDP, diastolic depolarization rate, maximal upstroke and repolarization velocities, and AP overshoot and amplitude and an increase in AP duration at 50% repolarization (APD_50_). Application (in separate experiments) of a lower 2-APB concentration of 1 μM also led to marked reduction in AP spontaneous rate (by 25.2 ± 6.2%; n = 6, *p* < 0.01), with slowing of diastolic depolarization, a more modest reduction in MDP and AP upstroke velocity, and overshoot (see Table 4).

**Figure 8:**
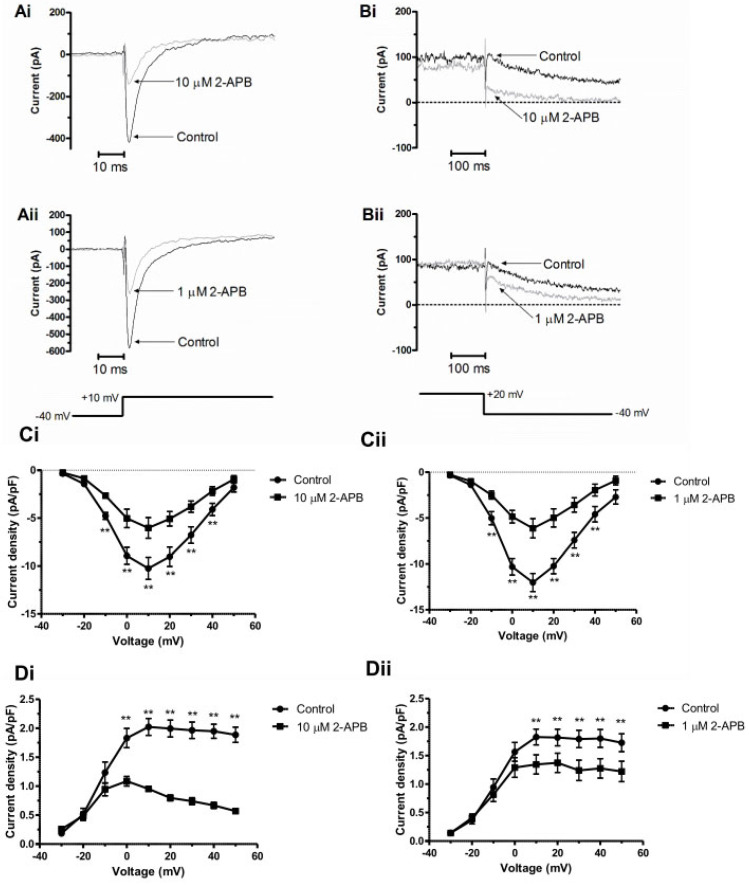
Effects of 2-APB on I_Ca,L_ and I_Kr_. (**A**) Traces in Ai and Aii show representative recordings of I_Ca,L_ at +10 mV, showing inhibition at both 10 µM (**Ai**) and 1 µM 2-APB (**Aii**); protocol is shown under (**Aii**). (**B**) Traces in Bi and Bii show representative recordings of I_Kr_ tails following activating commands to +20 mV, showing inhibition at both 10 µM (**Bi**) and 1 µM 2-APB (**Bii**); protocol is shown under **Bii**. (**C**) Plots in (**Ci**,**Cii**) show current–voltage (I–V) relationships for peak I_Ca,L_ elicited by 500 ms duration depolarizing voltage clamp commands from a holding potential of −40 mV. (**Ci**) shows effects of 10 µM 2-APB (n = 7) and (**Cii**) shows effects of 1 µM 2-APB (n = 8). (**D**) Plots in (**Di**,**Dii**) show current–voltage (I–V) relationships for I_Kr_ tail density at −40 mV following 500 ms activating commands to the potentials plotted. (**Di**) shows effects of 10 µM 2-APB (n = 7) and (**Dii**) shows effects of 1 µM 2-APB (n = 8). ** Significant differences between control and 2-APB values (2-way ANOVA with Bonferroni post hoc test).

The pronounced effects of 2-APB on AP amplitude and time course, particularly at 10 µM, raised a question as to whether the compound might exert direct effects on AVN ion channels beyond effects on IP_3_ signalling and hence reflect, for the purposes of this study, quite non-selective effects [29]. As both I_Ca,L_ and I_Kr_ are critical for AVN cell activity and the observed AP effects were consistent with their partial inhibition, we tested effects of 2-APB on these two currents. Recordings of I_Ca,L_ and I_Kr_ were made as described in Methods. Representative currents are shown in Figure 8(Ai,Aii,Bi,Bii): marked current reductions in both I_Ca,L_ and I_Kr_ were evident in the presence of both 1 and 10 µM 2-APB. Mean current–voltage relationships for the two currents are included in Figure 8(Ci,Cii) for I_Ca,L_ and Figure 8(Di,Dii) for I_Kr_, demonstrating reductions in each current over a range of test potentials. The higher 2-APB concentration reduced I_Kr_ more strongly than the lower one, corresponding to the greater depolarization of MDP in AP recordings in 10 than 1 µM 2-APB. The inhibition of these two currents in the presence of a calcium chelator in the patch pipette solution is suggestive of direct inhibitory effects of 2-APB at the concentrations employed, which confounds explanation of the compound’s effects on APs as being due to just IP_3_-R inhibition.

### 3.6. Effect of Photoreleased IP_3_ under AP Voltage Clamp

**Figure 9:**
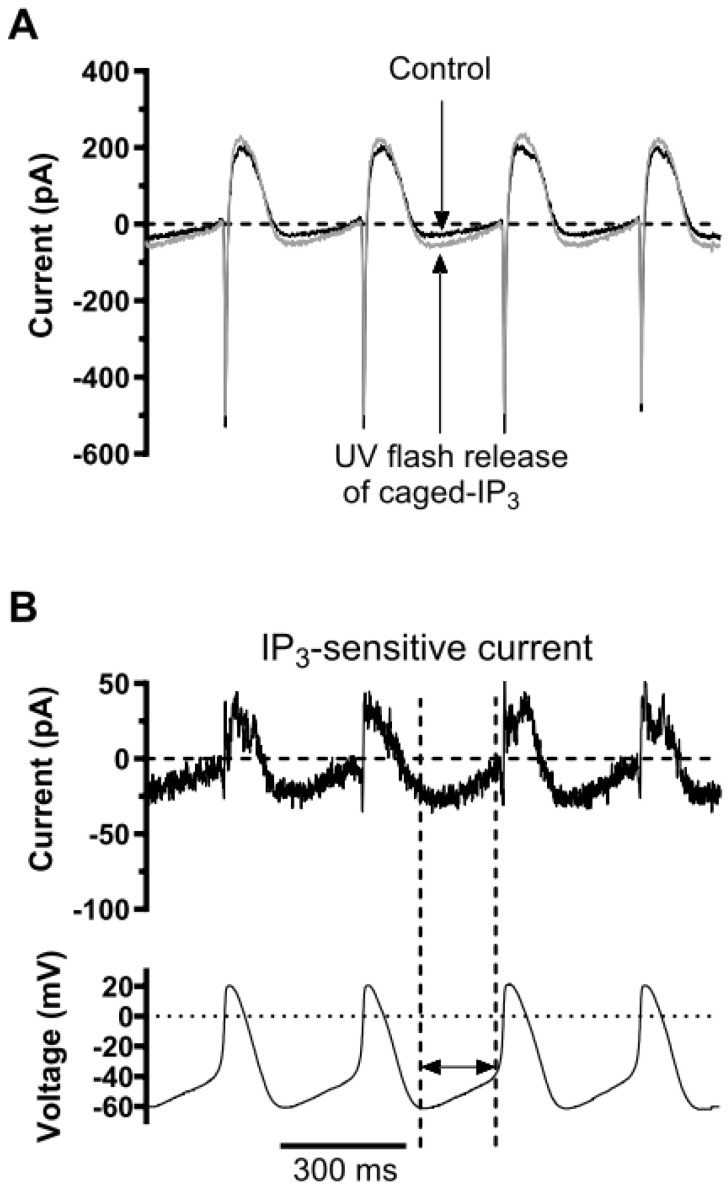
Effect of photoreleased IP_3_ on current during AP voltage clamp. (**A**) Superimposed net currents recorded from an AVN cell in control solution (black) and following repetitive UV excitation to photorelease caged IP_3_. Experimental protocol is shown vertically aligned with these traces in lower panel of (**B**). (**B**) IP_3_-sensitive current obtained by subtraction of control from UV-flash release current in ‘A’. Vertical dashed lines highlight the diastolic depolarization phase during one cycle of spontaneous activity. Note the inward IP_3_-sensitive current during this phase.

In a final series of experiments, we investigated the effect of repeated photostimulation under AP voltage clamp (Figure 9). A standardised template sequence of 4 AVN APs (lower panel of Figure 9B) was used as the voltage command, with the recording solutions as used for AP recording. The AP command series was applied first in control conditions and then following repeated application of UV flashes. Figure 9A shows superimposed currents in control and following photorelease of IP_3_, while Figure 9B shows the IP_3_-activated current (obtained by subtraction of control from +IP_3_ currents), aligned with the AP command series. As highlighted by the vertical dashed lines, an inward IP_3_-activated current was observed during the diastolic depolarization phase of the AP series. In six experiments, the mean maximal amplitude of this inward current was −0.56 ± 0.05 pA/pF. The reversal potential of this current was −35.4 ± 1.9 mV (n = 6). We also calculated the integral of the IP_3_-activated current during the diastolic depolarization phase (the time period from the MDP to the initiation of the AP upstroke phase) and found this to be −36.6 ± 3.3 fC/pF (n = 6). When comparable measurements were made without caged-IP_3_ in the patch pipette, the repeated flash-sensitive current integral during diastolic depolarization was −0.45 ± 3.7 fC/pF (n = 7; *p* < 0.01 vs. result with caged IP_3_). Thus, the inward current during diastolic depolarization was attributable to IP_3_ release and not UV excitation per se.

## 4. Discussion

### 4.1. IP_3_-R2s in the Rabbit AVN

It has been established that both atrial and ventricular myocytes express IP_3_-Rs, with IP_3_-R2 predominating over other isoforms and with higher atrial than ventricular IP_3_-R levels [30]. Co-staining of atrial myocytes for RyR2 and IP_3_-R2 showed subsarcolemmal IP_3_-Rs co-localised with RyRs [30]. In ventricular myocytes, IP_3_-R labelling exhibits z-line regularity and co-localisation with RyRs [34,35]. Atrial myocytes exhibit less regularity in IP_3_-R staining patterns [30,43]. Investigation of the mouse heart has shown the presence of mRNA for all three IP_3_-R types in pacemaker regions [42], with Western blot confirming expression of both IP_3_-R1 and IP_3_-R2 at the protein level in both SAN and AVN (with higher expression of IP_3_-R2) [42]. In murine SAN cells, IP_3_-R2 labelling showed some overlap with that for SERCA2a, which was used as an SR marker and exhibited a sarcomeric labelling pattern [42]. Subsarcolemmal IP_3_-R2 labelling that showed some co-localisation with RyR2 also detected in SAN cells [42]. Our data are broadly compatible with earlier findings and demonstrate partial overlap between RyR2 and IP_3_-R2 staining in AVN cells.

### 4.2. I_Ca,L_ and I_Kr_ Inhibition by 2-APB

2-APB was the first candidate for a membrane penetrant IP_3_-R inhibitor, with a reported IC_50_ for inhibition of IP_3_-induced Ca^2+^-release from cerebellar microsomal preparations of 42 µM [59]. 2-APB has been used to investigate atrial IP_3_-R signalling [36,37,38] and at low (2.5–5) µM concentrations to probe the role of IP_3_-R in the SAN [42,43]. It is known that 2-APB can exert effects on calcium-release activated current (I_CRAC_) [61] and members of the transient receptor potential (TRP) channel family [62]. However, under the conditions of the present study, 2-APB was found to inhibit I_Ca,L_ and I_Kr_ in experiments in which [Ca^2+^]_i_ was controlled by incorporation of BAPTA in the patch pipette. These effects occurred at concentrations that overlap those used to study IP_3_-R and are reminiscent of similar of inhibition of I_Ca,L_ and I_Kr_ from AVN cells by the cation channel inhibitor SKF-96365 [50]. 2-APB has been reported to activate members of the two-pore K^+^ channel family (TREK-1, TREK-2 and TRAAK) [63] and recently the Kv1.x family [64]. However, we are unaware of any previous report of inhibition of cardiac I_Kr_ or its molecular counterpart, hERG, by 2-APB. Due to the effects on I_Ca,L_ and I_Kr_ seen here, we are unable reliably to attribute slowing of spontaneous AP rate in AVN cells by 2-APB to inhibition of IP_3_-R alone. Significantly, our results with 2-APB extend the information available on non-IP_3_-R mediated effects of the compound and urge caution in its use to study the role(s) of IP_3_-R in modulating cardiac pacemaker cell excitability.

### 4.3. Evidence for Constitutive and IP_3_-R Activity in Modulating AVN Cell Rate

In contrast to our results with 2-APB, we observed no evidence for direct effects of Bt_3_-(1,4,5)IP_3_-AM on I_Kr_ and I_Ca,L_ under recording conditions comparable to those in which 2-APB was examined. Neither I_f_ nor I_NCX_ were directly affected by this intervention. It is therefore notable that Bt_3_-(1,4,5)IP_3_-AM application led to ~40% increase in spontaneous AP rate in our experiments, and in separate experiments also increased the rate of spontaneous Ca^2+^_i_ transients (by >30%). When applied to murine SAN cells, IP_3_-BM increased spontaneous Ca^2+^_i_ transient rate by 13% [42]. Direct, quantitative comparison of that finding with our study is difficult because of the different species employed. However, qualitative comparison leads to our conclusion that introducing cell-permeant IP_3_ accelerates the spontaneous rate of both SAN and AVN myocytes.

We are unaware of prior studies that have used XeC to study cardiac pacemaker tissue activity. Although XeC is a costly reagent, we applied it both externally (through pre-incubation) and via the patch pipette to ensure adequate exposure. This approach was serendipitous, as we observed little difference between the spontaneous rate of untreated myocytes and that of pre-treated AVN myocytes at commencement of recording. By contrast, once the whole-cell recording mode had been obtained, XeC entry into cells via the patch pipette was associated with a rapid and marked reduction in AP rate and slope of diastolic depolarization. That this effect is attributable to inhibition of IP_3_-Rs is supported by the observation that subsequent Bt_3_-(1,4,5)IP_3_-AM application to cells dialysed with XeC-containing pipette solution failed to accelerate the rate. Thus, our results with XeC support roles for constitutively generated IP_3_ and raised levels of IP_3_ in modulating AVN cell spontaneous AP rate.

A study of IP_3_ inhibitor effects on non-cardiac cells has claimed that heparin is more effective at inhibiting IP_3_-Rs than other inhibitors [65]. In additional experiments (not shown), we tested the effects of inclusion of heparin (5 mg/mL) in the pipette solution. This had a profound effect on AP morphology and produced a profound hyperpolarization of MDP, leading to quiescence in 8 out of 10 cells within 2 min. While this might be considered to be consistent with a major role for IP_3_-Rs in AVN cell pacemaking, the marked effects on multiple AP parameters and complete quiescence led us to consider that the use of heparin was problematic in this application.

Recent work on effects of IP_3_ release on atrial Ca^2+^ transients has highlighted the utility of the use of caged-IP_3_ [43]. Perhaps some of the most compelling evidence for IP_3_ modulation of spontaneous AP rate in AVN cells in the present study comes from the use of this approach: the extent of observed AP rate acceleration depended on the extent of UV stimulation supplied (and was independent of UV excitation alone). UV photolysis of caged-IP_3_ increased spontaneous AP rate by up to 30% after multiple flashes, which is similar to the effects of Bt_3_-(1,4,5)IP_3_-AM application. Under AP voltage clamp, the current activated by caged-IP_3_ was inwardly directed during the diastolic depolarization, explaining the slowing of AP rate in other experiments. While detailed investigation of the underlying basis for this current was beyond the scope of this investigation, in preliminary AP clamp experiments in which 20 µM nifedipine was applied, inward current was still observed during diastolic depolarization following release of IP_3_ from caged IP_3_.

### 4.4. Limitations, Future Work and Conclusions

Through adopting multiple approaches to the manipulation IP_3_ in AVN cells, this study demonstrates for the first time that IP_3_ modulates spontaneous AVN AP generation. Our data suggest roles for both constitutive IP_3_-R activation and IP_3_ changes in modulating spontaneous AP rate in pacemaking cells from this cardiac region. While this study constitutes important proof-of-concept evidence in this regard, our results leave open questions as to how AVN cell IP_3_ is changed and how consequent alterations in AP rate arise physiologically. For example, ET_A_ receptor activation is known to increase IP_3_ [66] and in murine SAN cells, application of 100 nM ET-1 increased spontaneous Ca^2+^_i_ transient rate and diastolic [Ca^2+^]_i_ [42].

As highlighted by others, responsiveness to intervention(s) that raise IP_3_ without G_q_-coupled receptor activation demonstrates effects of IP_3_ signalling that are independent of the activation of DAG (diacylglycerol) [43]. At 10 nM, ET-1 rapidly abolishes AVN cell spontaneous activity via activation of a tertiapin-Q sensitive K^+^ current [49]. Intriguingly, AVN cells rendered quiescent by ET-1 exhibit small-amplitude spontaneous membrane potential oscillations and it is conceivable that such events involve IP_3_-R mediated Ca^2+^_i_ mobilisation [49] and a balance between such an action and inhibitory effects of ET-1 might vary with ET-1 concentration. Future work is certainly warranted, both to reveal the mechanism(s) by which IP_3_ mobilisation accelerates spontaneous AVN rate and to investigate the role of G-protein-coupled receptor activation in AVN cells in mobilizing IP_3_ to modulate AVN excitability.

In this study, we focused on spontaneous AP measurement with only one series of experiments involving measurement of [Ca^2+^]_i_. The rationale for this was twofold: (i) AP measurements monitor directly an electrophysiological end point and (ii) omission of a Ca^2+^ fluorophore allows interrogation of IP_3_ effects without potential Ca^2+^_i_-buffering effects due to introduction of a Ca^2+^ indicator: the slower spontaneous rate of Ca^2+^_i_ transients (Figure 4) than of spontaneous APs in whole-cell recording seems to vindicate this decision. Nevertheless, having established that IP_3_ modulates spontaneous AP activity in AVN cells, it would be desirable for future studies to incorporate extensive [Ca^2+^]_i_ measurements, particularly as these would enable scrutiny of subcellular mechanisms of IP_3_ action in the AVN (cf. [28,42]). Our AP clamp experiments demonstrate that increasing intracellular IP_3_ activates an inward current during diastolic depolarization. The results of our experiments on Bt_3_-(1,4,5)IP_3_-AM suggest a lack of direct effect of IP_3_ on AVN cell I_NCX_, but they do not preclude an indirect effect in which increased [Ca^2+^]_i_ activates inward I_NCX_. While the most likely candidate for IP_3_ activated inward current may be I_NCX_ [24,26,27], this cannot be confirmed without direct experimental evidence, and future work is required to determine the identity of this current. In studying effects of 2-APB on AVN I_Ca,L_ and I_Kr_, we identified off-target effects of the compound that confound its use for studying IP_3_-R in this cardiac cell type. We did not study effects of 2-APB on other ionic currents, however, and cannot preclude the possibility that 2-APB may exert additional non-selective effects on AVN cells.

## 5. Conclusions

This is the first experimental investigation to report functional evidence for a role of IP_3_ in AVN. Our key conclusion is that IP_3_ can modulate AVN cell excitability: interventions that increase intracellular IP_3_ were found to increase AVN cell spontaneous AP rate, while interventions expected to inhibit IP_3_-R were found to decrease AVN cell spontaneous AP rate. This information advances our knowledge of the electrophysiology of this region of the heart and lays a foundation for future work. This study also provides new evidence for cardiac I_Ca,L_ and I_Kr_ inhibition by 2-APB, which adds to accumulating evidence that 2-APB has severe limitations as a tool with which to study cardiac IP_3_-Rs.

## Figures and Tables

**Table 1:** Effects of 10 µM Bt_3_-(1,4,5)IP_3_-AM on spontaneous action potentials in rabbit AVN cells.

Parameter	Control	10 µM Bt_3_-(1,4,5)IP_3_-AM
Spontaneous AP rate (beats s^−1^) (Percentage increase %)	2.85 ± 0.17	3.96 ± 0.21 ** (40.5 ± 5.7%)
Slope of pacemaker diastolic depolarization (mV s^−1^)	64.6 ± 8.4	102.3 ± 10.6 *
Maximal upstroke velocity (V_max_, V s^−1^)	4.67 ± 0.80	3.89 ± 1.14
Maximal repolarization velocity (V_rep_, V s^−1^)	−1.22 ± 0.08	−1.09 ± 0.08
AP duration at 50% repolarization (APD_50_, ms)	77.4 ± 3.5	73.3 ± 3.8
Maximal diastolic potential (MDP, mV)	−51.8 ± 2.8	−49.4 ± 1.9
Overshoot (mV)	20.6 ± 2.1	11.6 ± 2.9 *
AP amplitude (mV)	71.5 ± 4.0	61.0 ± 4.4

APs were recorded using whole-cell patch clamp in control conditions and following ~2 min of exposure to Bt_3_-(1,4,5)IP_3_-AM. Comparisons were made using a paired *t*-test: * *p* < 0.05, ** *p* < 0.01 vs. control; (means ± SEM; n = 10).

**Table 2:** Effects of 5 µM xestospongin C on spontaneous action potentials in rabbit AVN cells.

Parameter	Untreated Cells	Xestospongin C (Start of Recording)	Xestospongin C (~1 min of Recording)
Spontaneous AP rate (beats s^−1^) (Percentage decrease %)	2.85 ± 0.17	2.63 ± 0.14 (7.7%, vs. untreated cells)	1.94 ± 0.17 * ^##^ (23.1 ± 8.7%, vs. Xe-C At Start)
Slope of pacemaker diastolic depolarization (mV s^−1^)	64.6 ± 8.4	97.8 ± 13.2	51.0 ± 5.2 **
Maximal upstroke velocity (V_max_, V s^−1^)	4.67 ± 0.80	8.66 ± 1.30	8.47 ± 1.42 ^#^
Maximal repolarization velocity (V_rep_, V s^−1^)	−1.22 ± 0.08	−1.61 ± 0.16	−1.49 ± 0.12
AP duration at 50% repolarization (APD_50_, ms)	77.4 ± 3.5	84.7 ± 3.5	83.5 ± 3.1
Maximal diastolic potential (MDP, mV)	−51.8 ± 2.8	−53.4 ± 2.1	−57.6 ± 2.3 **
Overshoot (mV)	20.6 ± 2.1	31.8 ± 2.2	30.5 ± 2.2 ^##^
AP amplitude (mV)	71.5 ± 4.0	85.2 ± 4.0	88.1 ± 4.3 ^#^

APs with XeC containing pipette solution were recorded using whole-cell patch clamp immediately after gaining whole-cell access and ~1 min after gaining access (n = 11). For comparison, AP parameters for cells untreated with XeC (same data as Table 1 control, n = 10) are included. Paired *t*-test: * *p* < 0.05, ** *p* < 0.01, Xe-C after ~1 min vs. Xe-C at start; group *t*-test: ^#^ *p* < 0.05, ^##^ *p* < 0.01, Xe-C after ~1 min vs. untreated cells.

**Table 3:** Effects of UV flash photolysis liberation of caged IP_3_ on spontaneous action potential rate in rabbit AVN cells.

Parameter	Control	With UV Excitation of Caged IP_3_
Spontaneous AP rate (beats s^−1^) (Percentage increase %)	2.30 ± 0.37	2.90 ± 0.36 * (32.1 ± 9.5%)
Slope of pacemaker diastolic depolarization (mV s^−1^)	73.4 ± 17.6	110.5 ± 14.0 *
Maximal upstroke velocity (V_max_, V s^−1^)	6.55 ± 1.49	7.73 ± 1.66
Maximal repolarization velocity (V_rep_, V s^−1^)	−1.86 ± 0.15	−1.82 ± 0.17
AP duration at 50% repolarization (APD_50_, ms)	69.1 ± 5.1	71.0 ± 4.5
Maximal diastolic potential (MDP, mV)	−65.8 ± 1.9	−65.3 ± 2.3
Overshoot (mV)	26.1 ± 4.5	27.9 ± 4.4
AP amplitude (mV)	91.9 ± 4.5	93.2 ± 4.3

APs were recorded using whole-cell patch clamp in control conditions and following repetitive application of UV flash excitation to release IP_3_ from caged IP_3_ (100 µM in pipette solution). Comparisons were made using a paired *t*-test: * *p* < 0.05, control vs. UV flash (mean ± SEM; n = 7).

**Table 4:** Effect of 2-APB on spontaneous action potentials from rabbit AVN cells.

Parameter	Control	10 μM 2-APB	Control	1 μM 2-APB
Spontaneous AP rate (beats s^−1^) (Percentage decrease %)	3.10 ± 0.36	2.28 ± 0.37 ** (28.0 ± 4.3%)	2.78 ± 0.22	2.07 ± 0.22 ** (25.2 ± 6.2%)
Slope of pacemaker diastolic depolarization (mV s^−1^)	79.2 ± 18.9	24.6 ± 7.7 **	82.0 ± 9.2	34.8 ± 3.6 **
Maximal upstroke velocity (V_max_, V s^−1^)	5.50 ± 1.05	0.78 ± 0.12 **	7.03 ± 2.14	4.55 ± 1.54 **
Maximal repolarization velocity (V_rep_, V s^−1^)	−1.34 ± 0.11	−0.66 ± 0.05 **	−2.49 ± 0.50	−2.43 ± 0.51
AP duration at 50% repolarization (APD_50_, ms)	68.4 ± 3.1	117.8 ± 11.6 **	75.2 ± 5.7	90.7 ± 5.0 **
Maximal diastolic potential (MDP, mV)	−51.8 ± 2.5	−38.9 ± 3.4 **	−62.7 ± 2.8	−58.2 ± 3.5 *
Overshoot (mV)	24.5 ± 3.5	−0.19 ± 3.1 **	23.0 ± 4.3	13.6 ± 5.2 **
AP amplitude (mV)	76.3 ± 5.5	38.7 ± 3.0 **	85.5 ± 6.6	71.8 ± 8.4 **

APs were recorded using whole-cell patch clamp in control conditions and in the presence of the two concentrations of 2-APB shown (10 µM, n = 7; 1 µM, n = 6). Note each concentration has its own paired control. Comparisons were made using a paired *t*-test: Paired *t*-test: * *p* < 0.05, ** *p* < 0.01 vs. control.

## Data Availability

All the research data are available in the article.
